# Determinants of preventive behavior against Covid-19 in secondary school students based on Health Belief Model (HBM): a structural equations modeling (SEM)

**DOI:** 10.1186/s41043-024-00589-1

**Published:** 2024-06-26

**Authors:** Mohammad Saeed Jadgal, Mehrdad Karimi, Hadi Alizadeh-Siuki, Fatemeh Kord Salarzehi, MoradAli Zareipour

**Affiliations:** 1 Department of Public Health, Chabahar University of Medical Sciences, Chabahar, Iran; 2 Department of Public Health, Khoy University of Medical Sciences, Khoy, Iran; 3 Department of Public Health, School of Health, Torbat Heydariyeh University of Medical Sciences, Torbat Heydariyeh, Iran; 4 Department of Nursing, School of Nursing, Iranshahr University of Medical Sciences, Chabahar, Iran

**Keywords:** COVID-19, Preventive behavior, Health belief model, Students, Structural equation modeling

## Abstract

Measures such as education, improving knowledge, attitude and taking preventive action to protect against COVID-19 are vital strategies for prevention. The aim of this study was to determine the predictability of Health Belief Model (HBM) constructs in performing preventive behaviors against COVID-19 among secondary school students in Chabahar, Iran. In this cross-sectional-analytical study, 400 secondary school students of Chabahar city were investigated by simple random sampling. The data collection tool was a questionnaire including demographic characteristics, knowledge, behavior, and Health Belief Model constructs’ questions. Exploratory Factor Analysis (EFA) was used to evaluate the validity of HBM constructs, and the structural equation modeling (SEM) method was used to evaluate the direct and indirect effects of the relationship between knowledge, HBM constructs, and preventive behavior against COVID-19 based on the conceptual model. Based on the results of the structural modeling, the direct effect of knowledge on the constructs of the health belief model was positive and significant (β = 0.34, *P*-value < 0.001), and on the preventive behavior of students was insignificant (β = 0.12, *P*-value = 0.07) while the indirect effect of knowledge through increasing the constructs of the HBM on student behavior was positive and significant (β = 0.30, *P* < 0.001). The relationship between the constructs of the HBM constructs and student behavior was also positive and significant (β = 0.89, *P*-value < 0.001).Due to the fact that knowledge and HBM structures played a role in predicting the adoption of preventive behavior from COVID-19, it is possible to design appropriate interventions to increase knowledge, sensitivity, perceived severity, and self-efficacy, in order to recover from COVID-19 by adopting preventive behaviors.

## Introduction

The global epidemic of COVID-19, a severe acute respiratory syndrome, which emerged in December 2019, is considered a threat to the health and lives of millions of people around the world [1, 2]. The World Health Organization (WHO) has reported outbreaks of other acute respiratory diseases such as MERS and SARS in the last two decades. The death rate of SARS in 29 countries was 8,096, and the death rate of MERS in 27 countries was 858. Meanwhile, the number of deaths due to COVID-19 is much higher, so that the death due to this disease was 1870 people only in China until February 18th, 2020 [3]. And as of May 19th, 2020, 320,448 deaths from this disease have been reported in the whole world, despite the fact that COVID-19 has not yet been inhibited and the number of deaths caused by it is still increasing [4, 5]. Mortality from this disease varies according to age and other health conditions such as the history of underlying diseases [6, 7].

Measures such as education, improving knowledge and attitude, and taking preventive action to protect against COVID-19 are important strategies for prevention. Choosing an appropriate model to teach preventive behaviors is the first step in the health care program. One of the appropriate models for teaching preventive behaviors against COVID-19 is the Health Belief Model (HBM). The HBM emphasizes how a person’s perception increases his motivation to adopt preventive behaviors against illness. According to the HBM, in order to adopt preventive behaviors, a person must first face the problem, i.e. have a feeling of danger (perceived sensitivity), then understand the severity and seriousness of its complications (perceived severity), as well as receive positive symptoms from the environment (cues for action), believes the applicability of the disease prevention program (perceived benefits), find the factors preventing action to be less expensive than its benefits (perceived barriers) so that finally, take preventive measures against the disease. In addition, positive judgment about one’s abilities to adopt preventive behaviors (perceived self-efficacy) is also an accelerating force that causes a person’s need to adopt preventive behaviors [8]. One of the basic applications of this model is the primary prevention of a disease or an injury [9]. Globally, HBM has been used for years to prevent the risks of diseases and complications caused by them [10–12].

The health of students as future makers will play an important role in the future progression of the country [13, 14]. Therefore, ensuring the health of students is not only important in protecting them against COVID-19, but also in preventing the spread of the virus in society [15]. According to the decisions of the country’s Corona Disease Management Headquarters, students are currently using educational applications to continue their studies online, and there is a possibility of schools reopening and face-to-face exams in the near future [16]. On the other hand, according to epidemiologists, COVID-19 has an unknown future and there is a possibility that this pandemic will become a seasonal disease, and if the environmental conditions (gatherings in schools) exist, it can lead to serious consequences [17]. Considering the principle that students are one of the vulnerable groups against this disease, the presence of a disease in a school can cause an epidemic among students. Therefore, it seems important and necessary to use basic preventive measures and follow health protocols to reduce such risks in this group [18]. A review of the databases of Google Scholar, MagIran, Academic Jihad Scientific Information Database, Iran Medex, the Reference database of Islamic world sciences, Science Direct, PubMed, etc., published findings about the determinants of prevention of COVID-19 among academics, for students were not found in our country, so the present study was conducted with the aim of determining the predictability of HBM structures in performing preventive behaviors against COVID-19 among secondary school students of Chabahar city.

## Materials and methods

In this cross-sectional-analytical study, secondary school students of Chabahar city were examined. The criteria for entering the study included: second high school students, access to WhatsApp and Telegram social platforms, and willingness to participate in the study. Not participating and incomplete completion of the questionnaire were considered exclusion criteria. According to the prevalence of 37% of the behavior, maintaining social distance as a preventive behavior of COVID-19 in the study of Khazaee Pool et al. [19] and considering the error rate of 0.05%, alpha of 5%, the sample size was determined to be 358 people, for which 11% was added to the sample size to increase the accuracy of the study, and finally, 400 people were included in the study.

In the current research, simple random sampling was used in such a way that the link of the online questionnaire for the principals of second-secondary schools covered in Chabahar city was shared by the presenters on WhatsApp and Telegram, and subsequently, the principals of the schools shared the link of the questionnaire on the online channels of the covered students of the school, which was a member of the WhatsApp and Telegram groups. In this study, simple random sampling was used. For this purpose, among the 20 secondary high schools in the city, 10 high schools were selected by simple random sampling. And in the next step, according to the list of students’ names that was obtained from the principals of the target high schools, 40 secondary school students who met the criteria for entering the study were randomly selected from each high school.

After sending the link to the questionnaires to the principals of high schools, this link was sent by the principals to the target students through WhatsApp and Telegram platforms. The data collection tool in this research was a researcher-made questionnaire, which was used after confirming its validity and reliability. At the beginning of the electronic questionnaire, they were given sufficient explanations about the purpose and criteria for entering the study. The questionnaire designed in this research consisted of 3 sections, the first section included demographic characteristics, the second section included questions about knowledge and behavior, and the third section included questions about HBM constructs.

The scoring of the knowledge questions, which were in the form of yes/no and I don’t know, is that the “correct” answer was given a score of 3, the option “I don’t know” was given a score of 2, and the “wrong” answer was given a score of 1. Also, the behavior questions were in the form of “always”, “sometimes” and “never”. For this reason, the correct answer was given a score of 3, the sometimes answer was given a score of 2, and the wrong answer was given a score of 1. The questions of the constructs of the HBM also included a three-option Likert scale (agree/no opinion and disagree). For this purpose, a score of 3 was assigned to the correct answer, a score of 2 to the no opinion answer, and a score of 1 to the wrong answer.

### Statistical analysis

Mean ± SD for continuous variables and the frequency (percent) distribution of categorical data for responders have been reported as descriptive statistics. The EFA was employed to assess the construct validity of the Health Belief Model (HBM) constructs: susceptibility, severity, benefit, barrier, self-efficacy, and cues to action. The Principal Component Analysis (PCA) method was chosen for factor extraction due to its effectiveness in reducing data dimensionality and identifying patterns that represent the data well. The VARIMAX rotation, an orthogonal rotation method, was applied to achieve a simpler and more interpretable factor structure with greater variance. For model fit criteria, we relied on several indices to ensure a robust analysis: A) Kaiser-Meyer-Olkin (KMO) Measure was used to assess the sampling adequacy for each variable in the model and the complete model. A KMO value greater than 0.6 is considered acceptable to proceed with factor analysis, B)Bartlett’s Test of Sphericity was conducted to verify the appropriateness of factor analysis for our data set. A significant Bartlett’s test (*p* < 0.05) indicates that the variables are correlated well enough to provide a reasonable basis for factor analysis, C) Communalities initially set to 1 and the communalities were assessed after extraction to determine the amount of variance in each variable explained by the factors. Variables with low communalities were considered for removal to improve the model, D) Factor Loadings: A threshold of 0.4 was set for factor loadings, considering loadings above this value to be significant. This threshold helped in identifying items that strongly relate to each factor, ensuring that each construct is well-represented by its items.

### The structural model (SEM)

**Fig. 1:**
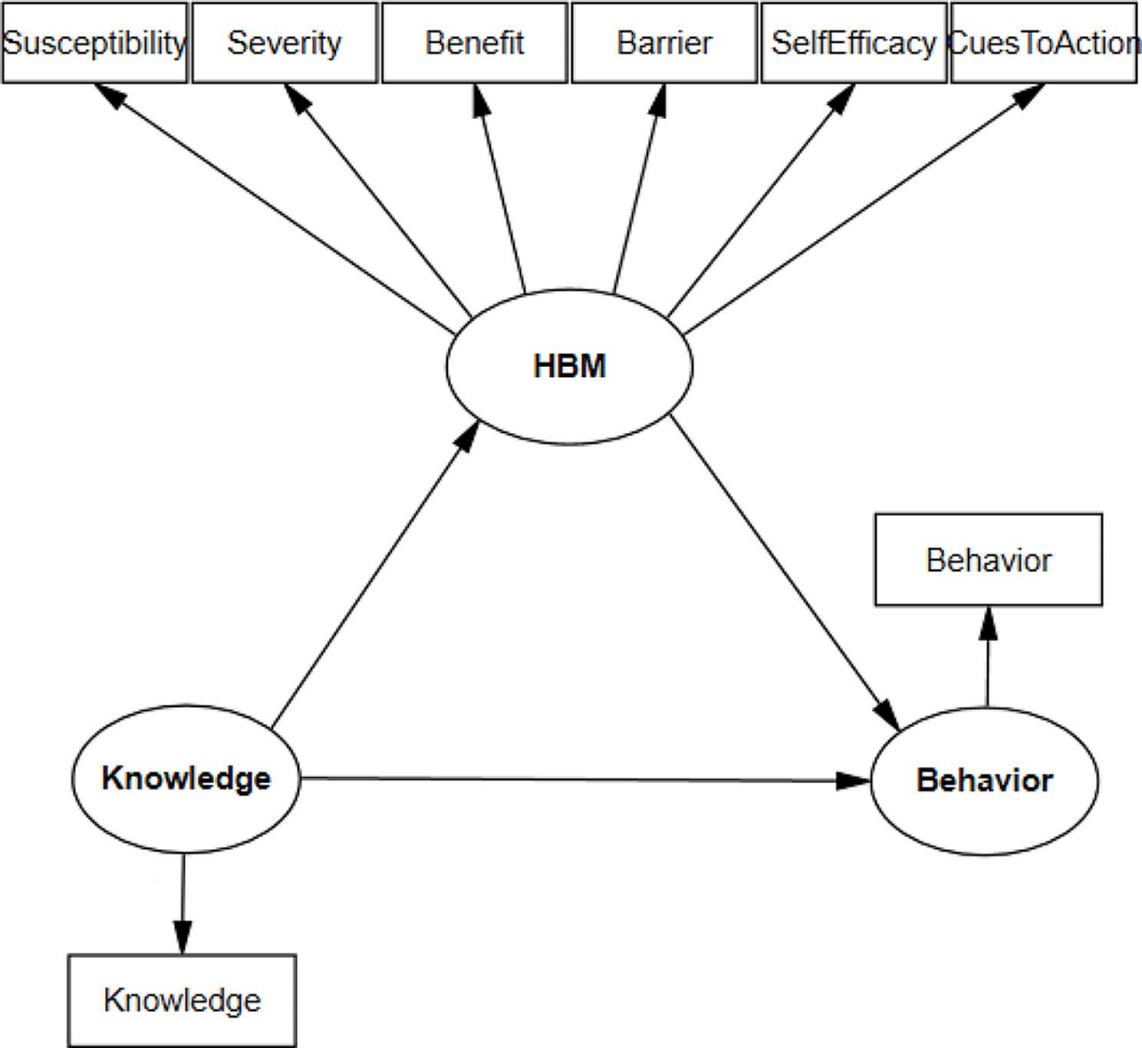
Conceptual model of the association among study variables

Structural Equation Modeling (SEM) was used to test hypothesized patterns of direct and indirect associations among knowledge, HBM and behavior constructs. Primary conceptual framework of the association among study variables are displayed in Fig. 1. Knowledge and behavior as latent variables have the role of exogenous independent and endogenous dependent variables, respectively. HBM construct have the role of mediator variable in the association between knowledge and behavior, and was considered as a latent variable which explained observed variables of susceptibility, severity, benefit, barrier, self-efficacy, and cues to action. In the following, the adjusted model has been introduced in which the gender, education of the student’s parents, and history of covid-19 in the students have been entered into the model as variables that moderate the relationship.

In our study, SEM models were estimated using the Maximum Likelihood Estimation (MLE) method. This method is widely recognized for its robustness and efficiency in parameter estimation when the assumptions of multivariate normality are met. The MLE approach is particularly advantageous for complex models, as it provides asymptotically unbiased, consistent, and efficient estimates.To evaluate the adequacy of our SEM models and so to assess how well the proposed models captured the covariance between all the measures, we employed a comprehensive set of fit indices, each offering unique insights into model performance: Some of the model fit indices and their acceptable thresholds [20, 21] assessed, included A) χ2 or chi-square test: This test assesses the discrepancy between the sample and fitted covariance matrices. A non-significant χ2 indicates a good fit; however, it is sensitive to sample size, leading us to consider additional indices B)

CMIN/DF: The ratio of the chi-square value to the degrees of freedom serves as a relative measure of model fit, with a value less than 3 indicating an acceptable fit, C) Comparative Fit Index (CFI) : This index compares the fit of our target model to an independent (null) model. Values greater than 0.90 suggest that the model adequately captures the data structure, D) Standardized Root Mean Square Residual (SRMR): This index represents the average discrepancy between the observed and predicted correlations. A value less than 0.10 is considered satisfactory., E) Root Mean Square Error of Approximation (RMSEA): This measure of fit per degree of freedom corrects for model complexity. A value less than 0.08 indicates a reasonable error of approximation in the population., E) Normed Fit Index (NFI), F) Incremental Fit Index (IFI), G) Goodness of Fit Index (GFI), and H) Adjusted Goodness of Fit Index (AGFI): These incremental fit indices compare the target model with a baseline model, with values greater than 0.90 indicating a good fit. The selection of these indices was guided by recommendations in the literature, which advocate for the use of multiple criteria to capture different aspects of model fit. By reporting a range of indices, we aim to provide a holistic view of the model’s performance. Data management, descriptive statistics, and comparisons were conducted using SPSS v25, while Amos v24 software was utilized to test the structural models. The significance level was set at *p* < 0.05, aligning with conventional standards for hypothesis testing.

## Results

Out of 400 secondary school students participating in this research, 69% were male and the rest were female. The majority of students reported that their father is illiterate (53.8) and their mother was of a primary level of education (45.5). 42.5% of the participating students had a history of contracting Covid-19 (Table 1). The mean and standard deviation of the main research variables are reported in Table 1.

**Table 1:** Base characteristics of study population

Variables	*n* (%), Mean ± SD
**Gender**	
male	276 (69.0)
female	124 (31.0)
**Father’s Education**	
illiterate	215 (53.8)
primary	97 (24.3)
guidance	73 (18.3)
high school & academic	15 (3.8)
**Mother’s Education**	
illiterate	80 (20.0)
primary	182 (45.5)
guidance	86 (21.5)
high school & academic	52 (13.0)
**Covid-19 history**	
no	230 (57.5)
yes	170 (42.5)
**Knowledge**	17.38 ± 3.75
**Susceptibility**	7.74 ± 2.86
**Severity**	7.18 ± 3.01
**Benefit**	6.78 ± 3.13
**Barrier**	6.84 ± 3.12
**Self-efficacy**	8.21 ± 3.71
**Cues to action**	5.50 ± 2.52
**Behavior**	14.16 ± 6.42

Data represented as frequency (percent) and Mean ± SD

### Reliability and validity of study scales

The results of EFA, including the standardized factor loadings of the items and the amount of explained variance to assess the construct validity of the scales, are reported in Table 2. Except for items 7 and 9 of the “knowledge” construct and item 2 of the “severity” construct, the rest have an acceptable factor loading (more than 0.50) and the explained variance of all factors is more than the acceptable limit of 50% and confirms the validity of the construct for the scales used in this research. Three items with low factor loading (know7, know8, severity2) were removed in the final factor analysis and the factor scores were calculated without taking into account the score of those items. Also, the value of the reliability coefficient using the internal consistency method (Cronbach’s alpha) for all scales is greater than 0.70 and shows acceptable reliability for the variables (Table 2).

**Table 2:** exploratory factor analysis results: factor loadings and explained variances

Scale and items	EFA	Alpha^#^
**Knowledge**	50.0*	**0.84**
Know1	0.685	
Know2	0.658	
Know3	0.758	
Know4	0.772	
Know5	0.630	
Know6	0.765	
Know7		
Know8	0.620	
Know9		
Know10	0.693	
**Susceptibility**	54.9*	**0.83**
Suscept1	0.786	
Suscept2	0.692	
Suscept3	0.783	
Suscept4	0.755	
Suscept5	0.738	
Suscept6	0.685	
**Severity**	65.0*	**0.85**
Severity1	0.598	
Severity2		
Severity3	0.832	
Severity4	0.830	
Severity5	0.862	
Severity6	0.876	
**Benefit**	77.3*	**0.93**
Benefit1	0.877	
Benefit2	0.882	
Benefit3	0.871	
Benefit4	0.895	
Benefit5	0.871	
**Barrier**	74.0*	**0.91**
Barrier1	0.852	
Barrier2	0.888	
Barrier3	0.852	
Barrier4	0.857	
Barrier5	0.852	
**Self-efficacy**	74.2*	**0.93**
SelfEfficacy1	0.849	
SelfEfficacy2	0.853	
SelfEfficacy3	0.875	
SelfEfficacy4	0.858	
SelfEfficacy5	0.866	
SelfEfficacy6	0.867	
**Cues to action**	79.4*	**0.88**
CuseToAction1	0.632	
CuseToAction2	0.976	
CuseToAction3	0.976	
CuseToAction4	0.933	
**Behavior**	71.5*	**0.95**
Behavior1	0.699	
Behavior2	0.883	
Behavior3	0.890	
Behavior4	0.872	
Behavior5	0.865	
Behavior6	0.842	
Behavior7	0.860	
Behavior8	0.849	
Behavior9	0.840	
Behavior10	0.837	

Note: the items of know6, know8 from knowledge scale and severity2 from severity scale excluded due to low factor loadings. The score of these scaled completed disregarding excluded items

* Explained variance

# Cronbach’s Alpha

### Correlation among study variables

The results of the correlation evaluation between research variables are shown in Table 3. According to Pearson’s correlation test, among HBM constructs, self-efficacy has the highest correlation with COVID-19 prevention behavior (*r* = 0.88, *P*-value < 0.001) and the lowest correlation with knowledge (*r* = 0.18, *P*-value = 0.015). The correlation between the knowledge and behavior scale was also positive and significant (*r* = 0.18, P-value < 0.001). The correlation between the behavior scale and HBM constructs was also positive and significant at the *P*-value < 0.001 level (Table 3).

**Table 3:** Pearson’s correlation test

Variables	1	2	3	4	5	6	7
1. knowledge							
2. susceptibility	0.28						
3. severity	0.12*	0.59					
4. benefit	0.18	0.60	0.90				
5. barrier	0.15	0.60	0.88	0.92			
6. self-efficacy	0.17	0.59	0.89	0.93	0.93		
7. cues to action	0.17	0.59	0.87	0.90	0.89	0.91	
8. behavior	0.18	0.57	0.75	0.79	0.78	0.88	0.76

* *P* < 0.05

Note: except for correlation between severity and knowledge, all other correlation coefficients significant at *P* < 0.001

### Structural model

#### Model 1. Unadjusted model

**Fig. 2:**
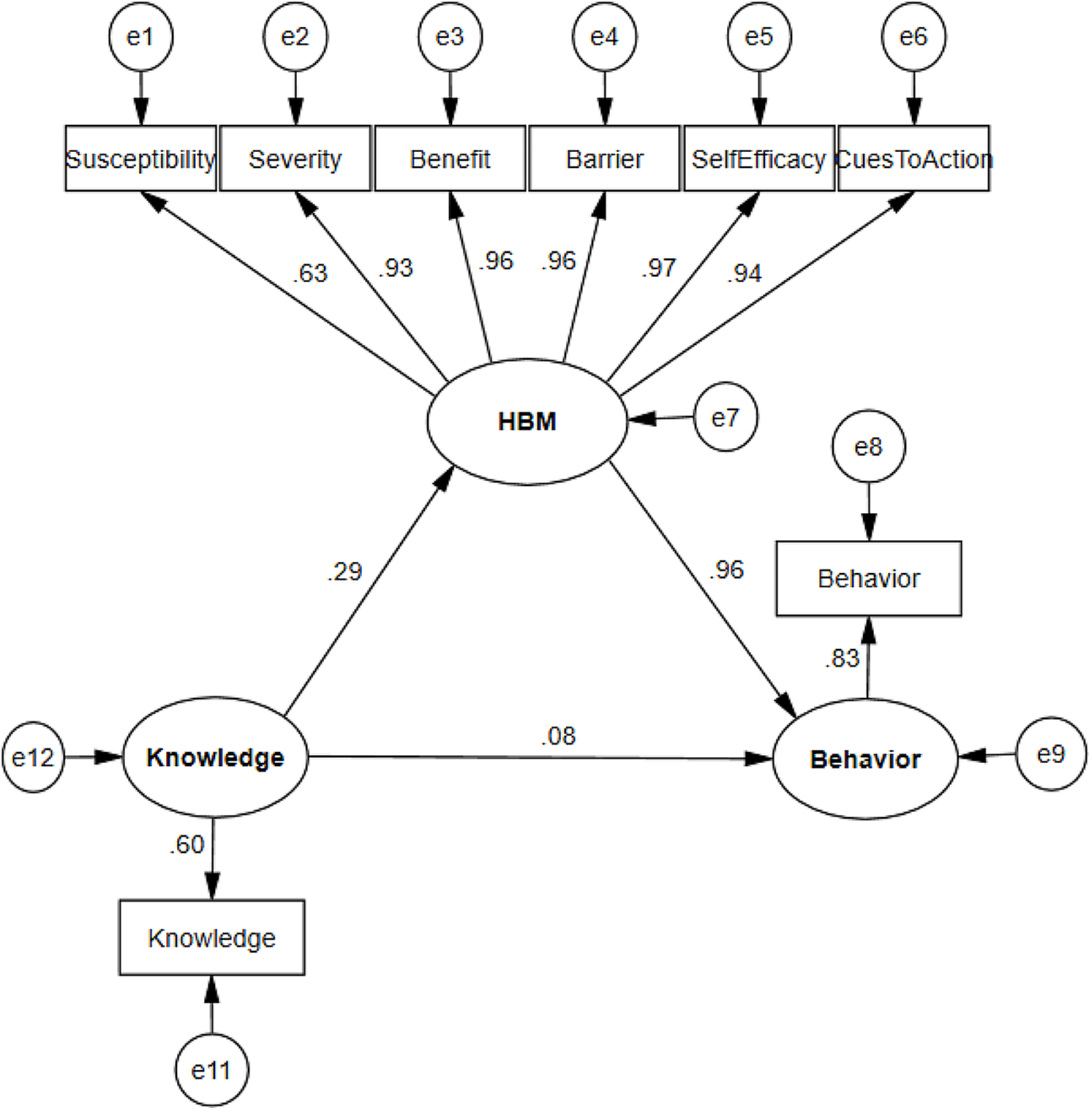
Unadjusted structural model pathway: standardized effects and factor loadings. Model.1 fit indices: chi-square=40.30, DF^1^ =19, chi-square to DF=2.12, SRMR^2^ =0.032, RMSEA^3^ =0.053, CFI^4^ =0.99, NFI^5^ =0.99, IFI^6^ =0.99, GFI^7^ =0.96, AGFI^8^ =0.93. ^1^Degree of Freedom. ^2^Standardized root mean square residual. ^3^Root mean square error of approximation. ^4^Comparative fit index. ^5^Normed fit index. ^6^Incremental fit index. ^7^Goodness of fit index. ^8^Adjusted goodness of fit index

The results of the unadjusted structural model which tests the interrelationship among research variables is shown in Fig. 2. The obtained fitted indices to evaluate the fit of the structural model are shown in the figure below, which shows the appropriate (acceptable) fit of the model. Based on the results of the unadjusted model, the direct relationship between knowledge and HBM constructs was positive and significant (β = 0.29, *P*-value < 0.001). The direct relationship between HBM constructs and behavior was also positive and significant (β = 0.96, *P*-value < 0.001). The direct relationship between knowledge and behavior was non-significant (β = 0.08, *P*-value = 0.21), but knowledge increases behavior indirectly through increasing HBM (β = 0.28, *P*-value = 0.014), which shows that HBM mediates the relationship between knowledge and behavior. In the context of Structural Equation Modeling (SEM), the construct validity of a latent variable is of paramount importance. The Health Belief Model (HBM) within our SEM framework is represented by six indicators, each demonstrating a factor loading greater than 0.5 and there are significant at *P* < 0.001. This is a strong indication of construct validity for several reasons: high factor loadings, convergent validity, reliability, theoretical justification, and practical significance. In conclusion, the HBM construct within this structural model is statistically and theoretically sound. The indicators’ high factor loadings not only confirm the construct’s validity but also enhance the robustness of the model, allowing for reliable predictions and interpretations.

### Model 2. Adjusted model

**Fig. 3:**
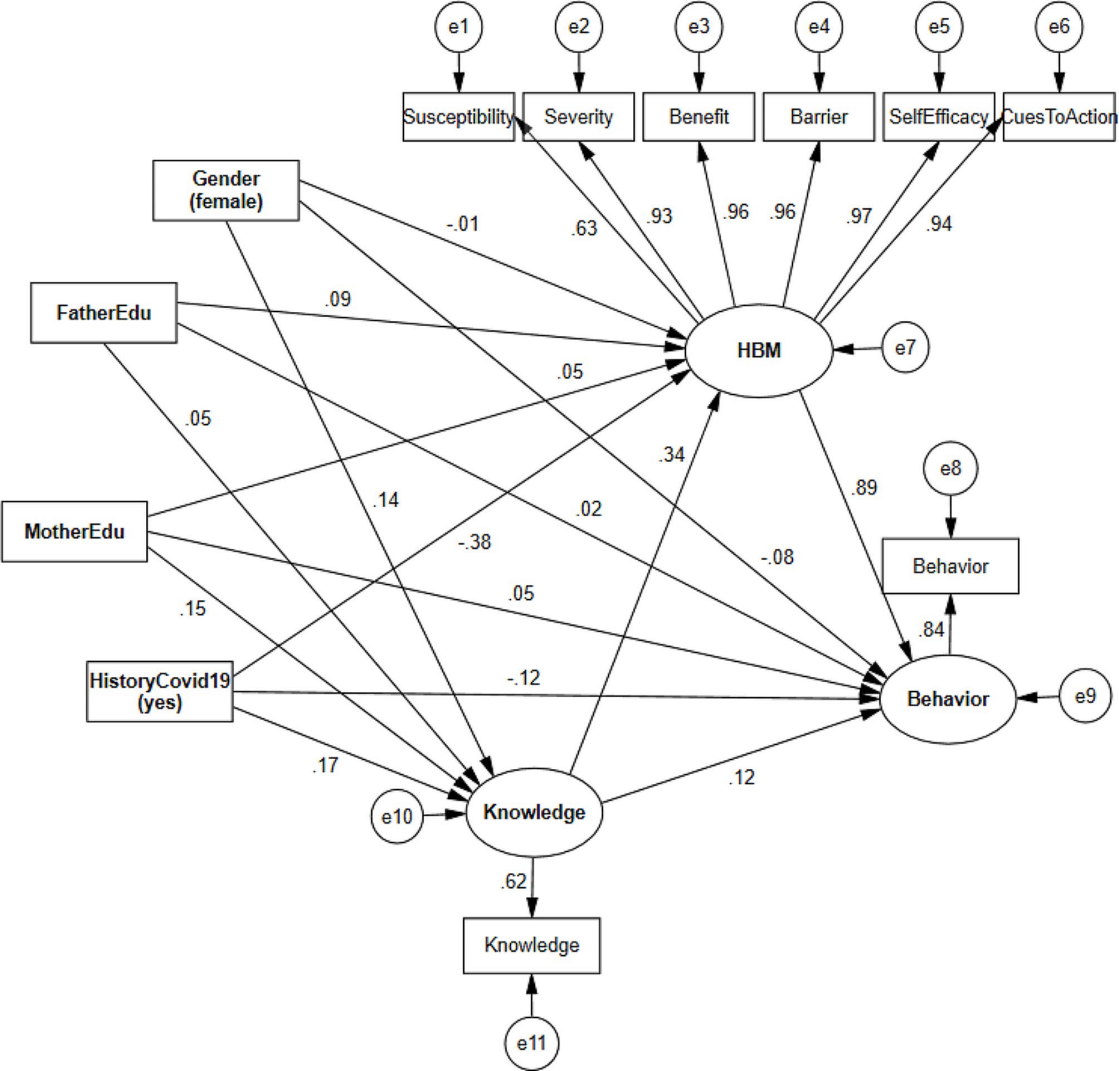
Adjusted structural model pathway: standardized effects and factor loadings. Model.2 fit indices: chi-square=102.04, DF=45, chi-square to DF=2.27, SRMR=0.041, RMSEA=0.056, CFI=0.99, NFI=0.97, IFI=0.99, GFI=0.96, AGFI=0.93

The results of tested adjusted structural model of the relationship among the research variables by adjusting the effect of students’ gender, their parents’ education, and the history of COVID-19 in students are shown in Fig. 3; Table 4. The obtained fitted indices to evaluate the fit of the adjusted structural model are shown in Fig. 3, which provides the goodness of fit for the model. Figure 3 presents the findings from a structural equation model. It shows the standardized direct and indirect effects of various exogenous independent variables on the mediators and endogenous dependent variables. The figure lists exogenous independent variables including gender, father’s education, mother’s education, and Covid-19 history and their effects on mediators including knowledge, HBM and endogenous dependent variable, Behavior.

**Table 4:** Adjusted structural model results: standardized direct and indirect associations

Variables		Direct effect		Indirect effect	
Independent →	Dependent	β	*P*-value	β	*P*-value
Gender (female)	Knowledge	0.14	0.04	-	-
	HBM	-0.02	0.77	0.05	0.03
	Behavior	-0.08	0.03	0.05	0.30
Father’s education	Knowledge	0.05	0.54	-	-
	HBM	0.09	0.08	0.02	0.38
	Behavior	0.02	0.53	0.10	0.03
Mother’s education	Knowledge	0.15	0.04	-	-
	HBM	0.05	0.31	0.05	0.04
	Behavior	0.05	0.21	0.11	0.01
Covid-19 history (yes)	Knowledge	0.17	0.03	-	-
	HBM	-0.38	< 0.001	0.06	0.02
	Behavior	-0.12	0.01	-0.27	< 0.001
Knowledge	HBM	0.34	< 0.001	-	-
	Behavior	0.12	0.07	0.30	< 0.001
HBM	Behavior	0.89	< 0.001	-	-

Based on the results of the fitted model (Table 4), In evaluating the direct relationship between gender and knowledge, behavior, and HBM constructs, female students had significantly more knowledge (β = 0.14, *P*-value = 0.04) and lower average behavior compared to male students (β=-0.08, *P*-value = 0.03) and direct relationship between gender and HBM constructs (β=-0.02, *P*-value = 0.77) was not significant, while the indirect relationship between gender and HBM constructs was significant and female students who had more knowledge led to an increase in the score of HBM constructs was compared to male students (β = 0.05, *P*-value = 0.03).

In context of direct relationship between parents’ education with the variables of the structural model, the father’s education did not have a significant direct relationship with the student’s knowledge, HBM, and behavior (*P*-value > 0.05) and indirectly had a significant positive relationship with the student’s behavior (β = 0.10, *P* = 0.03). The direct correlation of mother’s education with positive and significant with student knowledge (β = 0.15, *P*-value = 0.04) and the indirect correlation of mother’s education with HBM (β = 0.05, *P* = 0.04) and student behavior (β = 0.11, *P*-value = 0.003) was positive and significant.

The history of being afflicted with COVID-19 increases knowledge (β = 0.17, *P*-value = 0.03), decreases HBM constructs (β=-0.38, *P*-value < 0.001) and decreases student behavior (β=-0.12, *P*-value value = 0.001) directly. While a history of being afflicted with COVID-19 indirectly causes a significant increase in HBM (β = 0.06, *P*-value = 0.02) as knowledge increases. Getting infected with COVID-19 causes an increase in the student’s behavior score as knowledge increases, and as the HBM constructs reduce, leads to a (more) decrease in the behavior score. In general, the indirect effect of getting infected with COVID-19 on students’ behavior which is mediated by knowledge and HBM is negative and significant (β=-0.27, *P*-value < 0.001).

In the adjusted model, like the unadjusted model, the direct effect of knowledge on HBM constructs was positive and significant (β = 0.34, *P*-value < 0.001), and on student behavior was positive and non-significant (β = 0.12, *P*-value = 0.07), while the indirect effect of knowledge (by increasing HBM) on student behavior was positive and significant (β = 0.30, *P*-value < 0.001) and this result shows that HBM is a mediator or intermediate variable in the relationship between knowledge and student behavior. The relationship between HBM constructs and student behavior was also positive and significant (β = 0.89, *P*-value < 0.001).

## Discussion

Considering the unknown and new nature of the Coronavirus, it is necessary to use appropriate strategies to deal with this disease by public health authorities in the management of the communities and to create readiness in the people. In this regard, the use of psychological and behavioral models and theories plays an effective role in explaining the events and realities related to this disease. For this reason, the current study evaluated students’ risk perception and behavioral response to the spread of COVID-19 based on the constructs of the HBM. In the current study, the structures of the HBM had a positive correlation with each other, and with the preventive behavior of COVID-19, the highest correlation of the behavior with the self-efficacy construct, which was in line with the results of similar studies [22, 23]. It seems that due to the extensive information provided by the social and national media, and the notification of the Ministry of Health, people’s knowledge is higher than in the initial days of the COVID-19 outbreak and these factors lead to an increase in their perceived sensitivity and severity of being infected with COVID-19. On the other hand, understanding the benefits of doing preventive behaviors and the disadvantages of not doing so, along with the other mentioned cases, have led to increasing the efficiency of an individual regarding the positive belief of doing preventive behaviors from COVID-19 and their behavior has been affected and led to an increase in the level of preventive behaviors in COVID-19 affliction.

Investigating the direct and indirect relationship between gender and knowledge and HBM constructs showed that female students are more aware and have larger scores of HBM constructs compared to male students, as a result, they have taken more preventive behaviors against COVID-19, which satisfies the findings of other studies (Khafaie et al. [24] and Zareipour et al. [25]. The reason for this could be that girls are more responsible for their health. So that girls do their health examinations every year and are more willing to consult a physician and participate in health-related educational programs. Therefore, they are more concerned about their health and visit the physician when the first symptoms of a disease appear, and participate in their own treatment and in an educational program better than boys.

In evaluating the direct and indirect relationship between the education of the student’s parents and the variables of the structural model, each of the parent’s education, directly or indirectly, had a significant positive relationship with the knowledge, HBM constructs, and students’ behavior of preventing the COVID-19. In various studies, education is known as an important factor in preventive behaviors and health literacy [26–28]. The reason for the close relationship between education and health outcomes can be investigated from the three hypotheses of economic and work conditions, psychological and social resources, healthy lifestyles and good appetite. In the first hypothesis, educated people are less likely to remain unemployed and more likely to have high-paying full-time jobs. Based on the second hypothesis, educated people benefit from many psychological and social resources, such as a sense of self-control and high social support, as well as economic resources. Finally, according to the third hypothesis, educated people have a healthier lifestyle [29], which ultimately causes education to indirectly affect children’s health behaviors.

The findings of the study by Khazaee-Pool [19] and Kwok et al. [30], showed that the knowledge and HBM constructs of the studied subjects were high before affliction to COVID-19, such that most people stated that they were at risk of afflicting to COVID-19, and if so, they will experience severe symptoms and have high preventive behaviors. While in the present study, people with a history of COVID-19 had high knowledge, and the history of COVID-19 directly and indirectly, through the mediation of knowledge and HBM constructs, reduced preventive behavior in students.

Perhaps one of the reasons is that after getting another disease, they lost the feeling of being in danger (perceived sensitivity). Also, considering that most people have mild symptoms of COVID-19 and most people did not notice any complications after getting the disease, as a result, their perceived severity has decreased, and on the other hand, they are not interested in receiving information from the media and their perceived benefits and obstacles. Therefore, without understanding the severity of the symptoms of suffering from a disease and understanding the sensitivity of suffering from it, it will not lead to preventive behavior.

In the current study, the direct and indirect effects of knowledge on HBM constructs and HBM constructs on coronavirus prevention behaviors were positive. In Mirzaei et al.‘s study, HBM constructs were responsible for 29.9% of the variance of preventive behaviors against COVID-19 [31]. In the study by Khafaie et al. HBM constructs were able to predict 26% of the variance of preventive behaviors against COVID-19 [24], in Mahindarathne’s study, HBM model constructs predicted 49.7% of the variance of behavior [32]. Jadgal et al. regarding the use of HBM in predicting preventive behaviors reported similar results from tuberculosis disease [33]. All the mentioned studies are in line with the results of the present study and indicate the principle that for the formation of behavior, knowledge and having information alone is not enough, but sensitivity, severity, and self-efficacy and their pros and cons should be understandable for the individual and appropriate insight should be created to lead to a change in behavior.

Increasing people’s knowledge of coronavirus problems and complications increases their perception of severity and vulnerability to the disease. In addition, knowledge indirectly also affects the preventive behaviors of individuals through the influence on HBM structures. In other words, Knowledge First leads to improved perceptions of people, and then these perceptions affect preventive behaviors. The HBM model can therefore serve as a useful framework for designing and implementing educational interventions and promoting public awareness in the context of future pandemics. This can help increase preventive behaviors by improving people’s perceptions of severity, vulnerability, benefits and self-efficacy. The use of the HBM model can also help politicians and health officials analyze the factors influencing people’s preventive behaviors. Accordingly, Health politicians can design and implement targeted interventions and effectiveness to promote these behaviors in society. Overall, the health belief model is considered a useful tool for understanding and predicting preventive behaviors against similar epidemics in the future by providing a comprehensive framework of factors influencing health behaviors. Using this model can help manage and effectively control such diseases in the future.

## Conclusion

The results of the present study showed that boys were less careful in observing the limits and protecting themselves, the learning of self-care based on gender is reminded. People with a history of COVID-19 are exposed to more risks and injuries, and promotion and continuation of education in this group should be given special attention based on HBM constructs. Also, the findings showed the use of the health belief model in predicting preventive behavior from COVID-19, therefore, considering that knowledge and HBM constructs played a role in predicting the adoption of preventive behavior from COVID-19, it is possible to design appropriate interventions to increase knowledge. perceived sensitivity and severity and self-efficacy to improve preventive behaviors from COVID-19.

Due to the greater carelessness of boys in compliance with health cases, special training programs should be designed and implemented to promote awareness and self-care skills in boys in similar pandemics. In the design of educational interventions, attractive and appropriate methods can be used for different age and sexual groups. Also, for people with a history of coronavirus, strengthening training programs should be considered to increase their knowledge of the disease, emphasizing the need to follow prevention tips in possible future epidemics continuously. Finally, the health belief model (HBM) is used as a tool to design educational and informational interventions to promote preventive behaviors from similar viral diseases. These interventions can focus on increasing people’s knowledge of the disease, and their belief in the risk and severity of the disease, as well as strengthening their sense of self-efficacy in adhering to prevention tips. Continuous monitoring and evaluation of the effectiveness of interventions designed to improve future prevention behaviors of similar diseases is also importance very important.

## Data Availability

No datasets were generated or analysed during the current study.
